# Rates of Subjective Failure After Both Isolated and Combined Posterior Cruciate Ligament Reconstruction: A Study From the Norwegian Knee Ligament Registry 2004-2021

**DOI:** 10.1177/03635465241238461

**Published:** 2024-03-29

**Authors:** Gilbert Moatshe, Christopher M. LaPrade, Anne Marie Fenstad, Andreas Persson, Matthew LaPrade, R. Kyle Martin, Lars Engebretsen, Robert F. LaPrade

**Affiliations:** † Oslo University Hospital and University of Oslo, Oslo, Norway; ‡ Oslo Sports Trauma Research Center, Norwegian School of Sports Sciences, Oslo, Norway; § OrthoCarolina, Charlotte, North Carolina, USA; ‖ Norwegian Knee Ligament Registry, Haukeland University Hospital, Bergen, Norway; ¶ Department of Orthopaedic Surgery, Medical College of Wisconsin, Milwaukee, Wisconsin, USA; # Department of Orthopaedic Surgery, University of Minnesota, Minneapolis, Minnesota, USA; ** Department of Orthopedics, CentraCare, St Cloud, Minnesota, USA; †† Twin Cities Orthopedics, Edina, Minnesota, USA; Investigation performed at Oslo University Hospital, Oslo, Norway

**Keywords:** posterior cruciate ligament, posterior cruciate ligament reconstruction, registry, multiligament knee injury

## Abstract

**Background:**

Outcomes after posterior cruciate ligament (PCL) reconstruction (PCLR) have been reported to be inferior to those of anterior cruciate ligament reconstruction. Furthermore, combined ligament injuries have been reported to have inferior outcomes compared with isolated PCLR.

**Purpose/Hypothesis:**

The purpose of this study was to report on PCLR outcomes and failure rates and compare these outcomes between isolated PCLR and multiligament knee surgery involving the PCL. The hypothesis was that combined PCL injury reconstruction would have higher rates of subjective failure and revision relative to isolated PCLR.

**Study Design:**

Cohort study; Level of evidence, 3.

**Methods:**

Patients with primary PCLR with or without concomitant ligament injuries registered in the Norwegian Knee Ligament Registry between 2004 and 2021 were included. Knee injury and Osteoarthritis Outcome Score (KOOS) totals were collected preoperatively and at 2 years and 5 years postoperatively. The primary outcome measure was failure, defined as either a revision surgery or a KOOS Quality of Life (QoL) subscale score <44.

**Results:**

The sample included 631 primary PCLR procedures, with 185 (29%) isolated PCLR procedures and 446 (71%) combined reconstructions, with a median follow-up time of 7.3 and 7.9 years, respectively. The majority of patients had poor preoperative knee function as defined by a KOOS QoL score <44 (90.1% isolated PCLR, 85.7% combined PCL injuries; *P* = .24). Subjective outcomes improved significantly at 2- and 5-year follow-up compared with preoperative assessments in both groups (*P* < .001); however, at 2 years, 49.5% and 46.5% had subjective failure (KOOS QoL <44) for isolated PCLR and combined PCLR, respectively (*P* = .61). At 5 years, the subjective failure rates of isolated and combined PCLR were 46.7% and 34.2%, respectively (*P* = .04). No significant difference was found in revision rates between the groups at 5 years (1.9% and 4.6%, respectively; *P* = .07).

**Conclusion:**

Patients who underwent PCLR had improved KOOS QoL scores relative to their preoperative state. However, the subjective failure rate was high for both isolated and multiligament PCLR. Within the first 2 years after surgery, patients who undergo isolated PCLR can be expected to have similar failure rates to patients who undergo combined ligament reconstructions.

Posterior cruciate ligament (PCL) tears are often treated with surgical intervention, and surgical reconstruction has been reported to be cost-effective in comparison with nonoperative management.^19^ However, the current literature has reported variable results in terms of subjective clinical outcomes and objective side-to-side differences in posterior tibial translation on stress radiographs after PCL reconstruction (PCLR).^11,14,22,28-30^ In particular, the postoperative outcomes after PCLR and anterior cruciate ligament reconstruction (ACLR) have shown variable results; some studies have reported worse preoperative and postoperative outcomes for PCLR,^1,16,22^ whereas another reported no difference in outcomes postoperatively between isolated PCLR and isolated ACLR.^13^

Many reasons have been proposed in the literature for the poor results reported after PCLR. Biomechanical studies have demonstrated superior outcomes with double-bundle PCLR in comparison with single-bundle PCLR.^9,10^ Clinical study results have been mixed; double-bundle PCLR has been shown to be superior in some studies,^4,29^ whereas other studies have not found a difference between single- and double-bundle reconstructions.^30^ Double-bundle PCLR is less commonly performed than single-bundle PCLR; the Danish Knee Ligament Reconstruction Registry reported that <3% of PCLR procedures used a double-bundle technique.^16^ In addition, Noyes and Barber-Westin^18^ reported other risk factors for failure after PCLR, including posterolateral corner deficiency (40%), improper graft tunnel placement (33%), and associated varus malalignment (31%).

Previous studies from the Norwegian Knee Ligament Register (NKLR) have evaluated preoperative and short-term clinical results, as measured by the Knee injury and Osteoarthritis Outcome Score (KOOS), after ACLR^1,12,26^ and PCLR.^1,20^ However, at this time, no studies have yet evaluated the subjective failure rate or subsequent revision rate of PCLR using a national registry for either isolated PCL injury or multiligament knee injury involving the PCL. Multiligament injuries are more complex injuries compared with isolated knee ligament injuries. Limited data are available directly comparing isolated PCLR procedures versus musculoskeletal injury reconstructions; therefore, a study was performed to compare outcomes in isolated PCLR and combined PCLR.

The purpose of this study was to report on subjective outcomes and failure rates after PCLR and to compare these outcomes between isolated PCLR and multiligament knee injury involving the PCL. The hypothesis was that combined PCL injuries would have a higher subjective failure rate and revision rate in comparison with isolated PCL injuries.

## Methods

This was a retrospective review of prospectively collected data from a national registry. The manuscript was prepared according to the Strengthening the Reporting of Observational Studies in Epidemiology checklist.^27^

The NKLR was established in 2004 and is a nationwide registry that prospectively collects data on cruciate ligament surgeries from all hospitals and private clinics in Norway. The overall goal is to evaluate current practices and improve treatment outcomes. The registry is supported by the Norwegian government, and it has been mandatory to report data from private and public hospitals since 2017. The registry has been reported to have high rates of hospital compliance, with reported compliance rates of 86% to 97%, and high positive predictive values for key variables recorded for primary reconstructions.^6,17,31^

Detailed information regarding the procedure (date of injury and surgery, activity at the time of injury, concomitant injuries, graft used, graft fixation, intraoperative findings and procedures, and patient-specific data) are reported by the surgeon to the registry and have been described previously.^6^ Patient-reported outcomes are measured with the KOOS preoperatively and at 2-, 5-, and 10-year follow-up. Subsequent knee surgery to the index knee is also reported to the registry by the surgeon and linked to the primary reconstruction based on the patient's specific national identification number (social security number). Revision surgery is registered and reported differently from other subsequent surgeries, such as meniscal surgery in the registry, and can therefore be distinguished.

**Figure 1.**
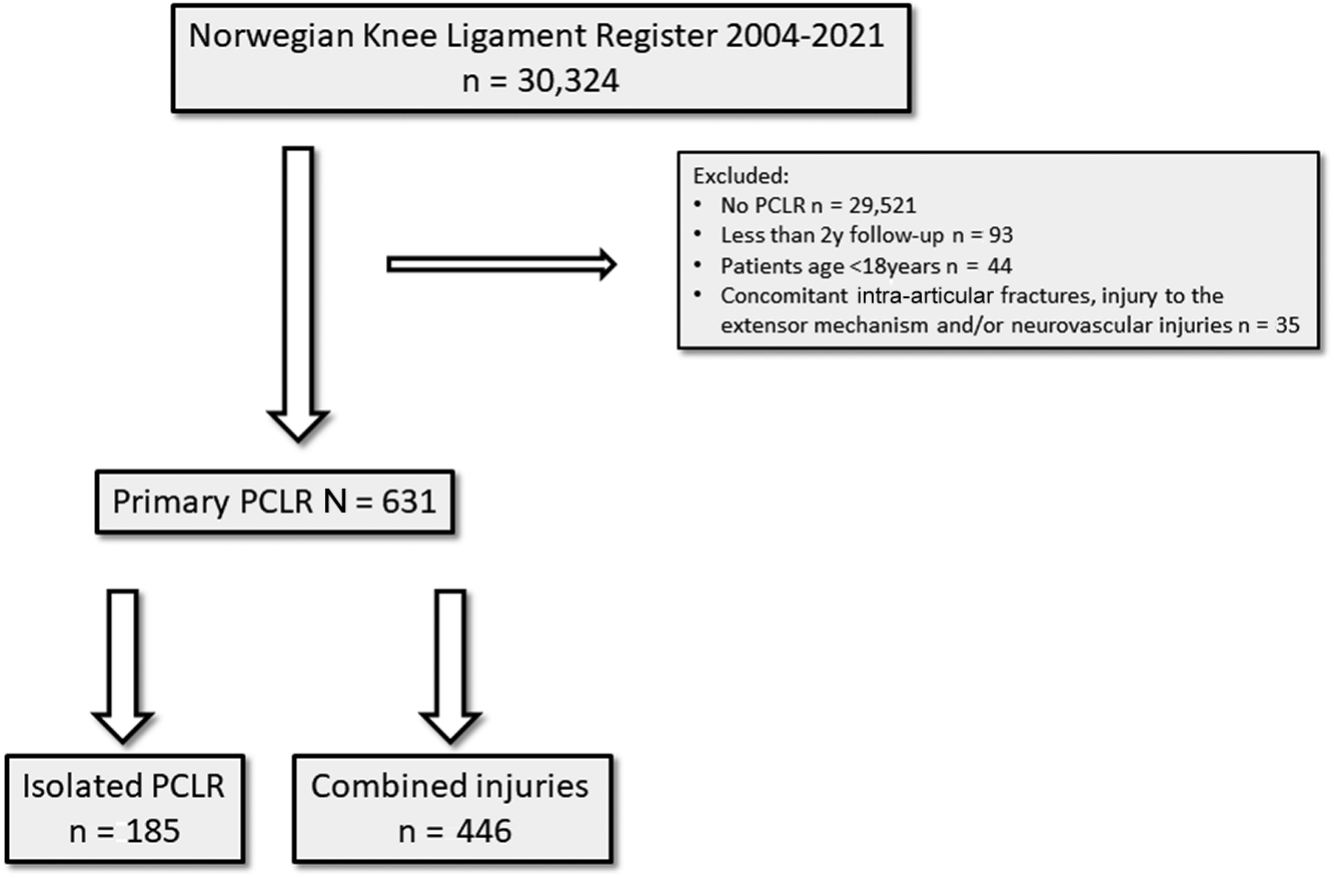
A flowchart illustrating the study cohort with included patients. PCLR, posterior cruciate ligament reconstruction.

All patients registered in the NKLR between June 2004 and December 31, 2021, were eligible for inclusion in this study. All patients who underwent primary PCLR with or without concomitant ligament injuries registered in the NKLR between 2004 and 2019 were included. Patients who were operated in 2019 had 2-year follow-up data are included in the analysis, but these patients are not included in the 5-year follow-up because the 5-year follow-up data were not yet available at the time of publishing of this study. Skeletally immature patients and minors <18 years (n = 44) and patients with concomitant intra-articular fractures, injury to the extensor mechanism, and/or neurovascular injuries (n = 35) were excluded (Figure 1).

The following variables were retrieved from the NKLR: patient age, sex, date of injury, date of primary surgery and potential revision surgery, activity at the time of injury, body mass index, meniscal injuries (yes/no), other reported ligamentous injuries (anterior cruciate ligament, medial and lateral collateral ligament, posterolateral corner) and potential reconstructions to those structures (yes/no), graft choice for PCLR and ACLR, cartilage injuries (including the International Cartilage Regeneration & Joint Preservation Society [ICRS] grade), and KOOS reported preoperatively and at 2- and 5-year follow-up. The variable “activity at the time of injury” was further classified into pivoting sports (handball, soccer, basketball, and floorball), skiing, other sports, and other. Patient age, sex, time from injury to surgery, meniscal injury (yes/no), and cartilage injury (no injury, ICRS 1-2, ICRS 3-4, missing data) were included in the statistical analysis as possible confounding factors.

Patients were considered to have an isolated PCLR when no injuries or reconstructions were reported to other knee ligaments. Patients were considered to have a multiligament reconstruction when ≥1 of the other main knee ligaments was reconstructed in addition to the PCL. The primary outcome measure was failure, defined as either a revision surgery or a KOOS Quality of Life (QoL) subscale score <44. The KOOS QoL subscale was chosen as an indicator of subjective failure after PCLR. The KOOS QoL subscale has been considered previously an indicator of subjective failure after ACLR when <44, which corresponds with a more than moderately decreased knee-related quality of life.^5,7^

### Statistical Analysis

All statistical analyses were performed using SPSS Statistics, Version 26.0.1.0 (IBM Corp) and R Version 4.0.2 (R Centre for Statistical Computing). The Pearson chi-square test was used for comparison of categorical variables and Student *t* test for numeric measures. The nonparametric independent-samples median test was used when the distribution was asymmetrical or not Gaussian. A nonparametric independent-samples test was used to investigate the possible difference in the KOOS QoL score between the groups preoperatively and at 2 and 5 years after treatment. Survival time for the 2 subgroups of patients was calculated using Kaplan-Meier estimates. Endpoint was revision of any cause. Multivariate logistic regression analyses were used to calculate odds ratios between the subgroups, with adjustments for possible confounding of sex, age, meniscal injury, cartilage injury, and activity at the time of injury. All tests were 2-sided, and *P* values <.05 were considered statistically significant. Revision rates at 2 and 5 years were calculated using Kaplan-Meier analysis, and crude 2-year revision rates with 95% CI were reported.

## Results

### Patient Characteristics

After applying inclusion and exclusion criteria, we included 631 patients with PCL injuries in the final analysis. Of these, 185 patients had an isolated PCLR and 446 patients underwent multiligament knee reconstructions involving the PCL. Patients who underwent isolated PCLR were significantly younger (median age 28 vs 39 years; *P* < .001), had a lower body mass index (*P* = .008), and had sustained injury during pivoting sports at a significantly higher rate compared with those who underwent multiligament reconstruction (Table 1). More patients had sustained multiligament injuries than isolated PCL injuries during skiing. The median time to surgery was significantly shorter for multiligament reconstruction compared with isolated PCLR, and a higher percentage of male patients and those with meniscal injury had sustained a multiligament knee injury (Table 1).

**Table 1:** Study Population Characteristics (N = 631)^*a*^

Factors	Isolated PCLR (n = 185)	Multiligament Reconstruction (n = 446)	*P*
Age, y, median (IQR)	28 (18)	39 (22)	<.001^*b*^
Age group, n (%)			**<.001**
0-19 y	40 (21.6)	31 (7.0)	
20-29 y	64 (34.6)	112 (25.1)	
30-44 y	59 (31.9)	151 (33.9)	
≥45 y	22 (11.9)	152 (34.1)	
Male patients, %	59.5	64.1	.270
Activity at the time of injury, n (%)			**<.001**
Pivoting sports	56 (30.3)	52 (11.7)	
Skiing	16 (8.6)	151 (33.9)	
Other sports	5 (2.7)	14 (3.1)	
Other	108 (58.4)	229 (51.3)	
Concomitant ligament injuries not reconstructed, n			
ACL	—	2	
MCL	—	44	
LCL	—	14	
PLC	—	21	
Graft choice for PCLR, n (%)			**<.001**
BPTB	13 (7.0)	14 (3.1)	
Hamstring	121 (65.4)	195 (43.7)	
Allograft	44 (23.8)	151 (33.9)	
Other/unknown	7 (3.8)	86 (19.3)	
Graft choice for ACLR (n = 349), n (%)			
BPTB	—	237 (67.9)	
Hamstring	—	68 (19.5)	
Allograft	—	35 (10.0)	
Other/unknown	—	9 (2.6)	
Cartilage injury, n (%)^*c*^			.055
No reported injury	133 (71.9)	283 (63.6)	
ICRS 1-2	26 (14.1)	99 (22.2)	
ICRS 3-4	26 (14.1)	63 (14.2)	
Meniscal injury, n (%)			**<.001**
No injury reported	157 (84.9)	311 (69.7)	
Meniscal injury	28 (15.1)	135 (30.3)	
Treatment for meniscal injury, n			
No treatment	4	17	
Partial resection	7	35	
Total resection	0	1	
Resection not specified	5	26	
Suture	11	49	
Transplant	0	1	
Unknown	1	6	
Body mass index, mean ± SD^*d*^	26.2 ± 4.4	27.6 ± 4.6	.008
Time to surgery from injury, mo, median (IQR)^*e*^	19.4 (36.2)	5.9 (13.1)	**<.001**
Surgical time, min, median (IQR)^*f*^	90 (45)	163 (82)	**<.001**
Follow-up time, y, median (IQR)	7.3 (9.3)	7.9 (8.6)	.349
Patients at risk, n (%)			
2-year follow-up	157 (84.9)	391 (87.7)	
5-year follow-up	125 (67.6)	307 (68.8)	

a Boldface indicates statistical significance. Dashes indicate not applicable. ACL, anterior cruciate ligament; ACLR, anterior cruciate ligament reconstruction; BPTB, bone–patellar tendon–bone autograft; ICRS, International Cartilage Regeneration & Joint Preservation Society; LCL, lateral collateral ligament; MCL, medial collateral ligament; PCLR, posterior cruciate ligament reconstruction; PLC, posterolateral corner.

b Independent-samples median test (nonparametric).

c Missing ICRS classification in 1 patient having multiligament reconstruction.

d Available data on 461 patients (73.1%); isolated PCLR, n = 146 (78.9%); multiligament reconstruction, n = 315 (70.6%).

e Available data on 612 patients (97.0%), independent-samples median test (nonparametric).

f Available data on 626 patients (99.2%), independent-samples median test (nonparametric).

### Outcomes

**Figure 2.**
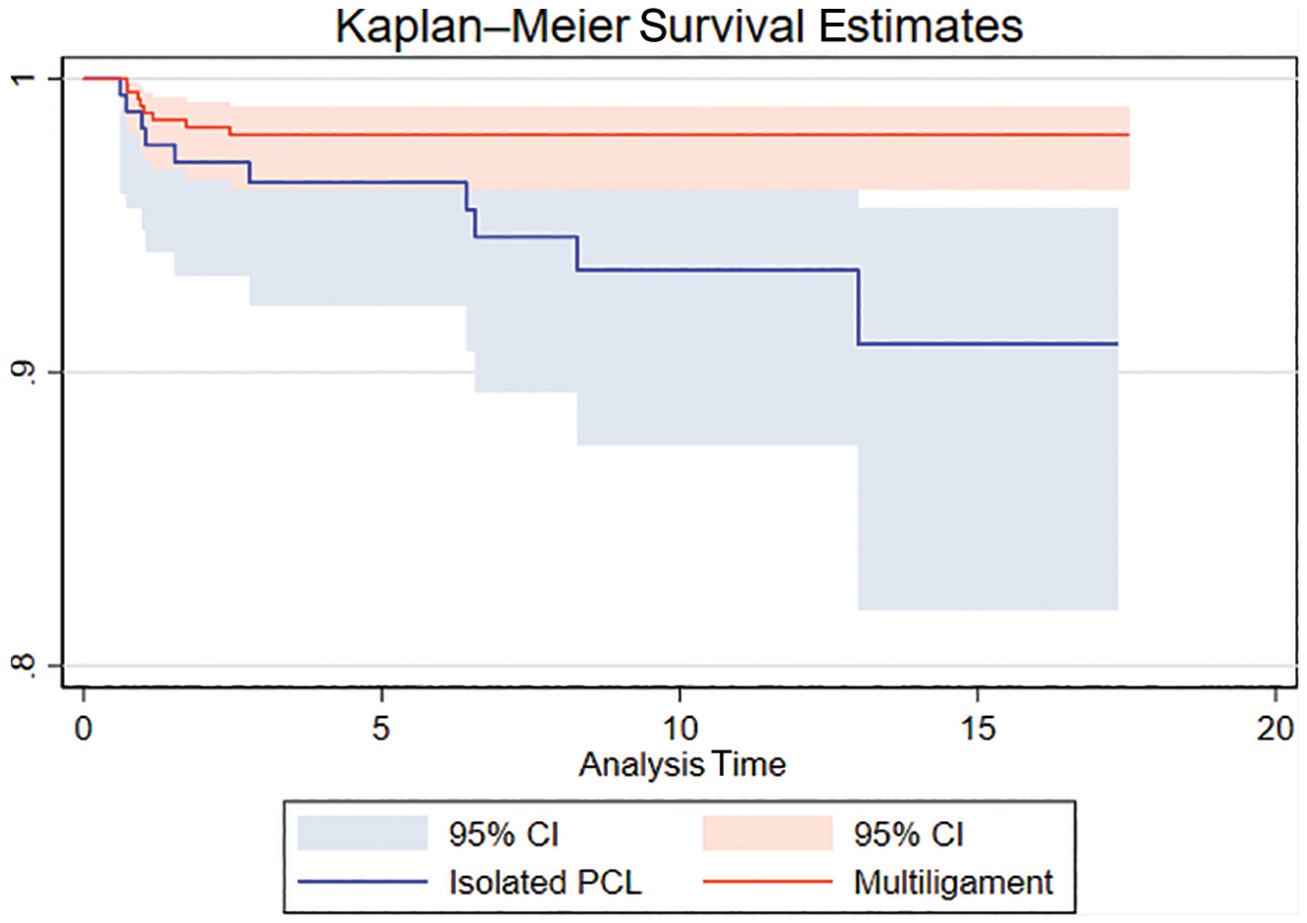
Revision survival curves for the groups undergoing isolated posterior cruciate ligament (PCL) reconstruction (lower) and multiligament PCL reconstruction (upper).

The proportion of patients with subjective failure (KOOS QoL <44) decreased from preoperative to postoperative assessment in both groups (Table 2 and Figure 2). The improvement in KOOS QoL for the 2 groups from the preoperative assessment to 2- and 5-year follow-up was statistically significant (*P* < .001). No significant difference was found in the proportion of patients with subjective failure preoperatively and at 2-year follow-up between isolated PCLR and PCLR with multiligament reconstruction. The proportion of patients with subjective failure preoperatively and at follow-up points are presented in Table 2. Mean scores for all KOOS subscales are presented in Table 3. At 2-year follow-up in the isolated PCLR and PCL-based multiligament reconstruction, 49.5% and 46.5% of patients had subjective knee failure, respectively. The proportion of subjective failures (KOOS QoL <44) did not change between 2 years and 5 years in the isolated PCLR group (*P* = .85); however, in the multiligament reconstruction group, the proportion of failures decreased between 2- and 5-year follow-up (*P* = .003). The proportion of patients with KOOS QoL <44 was significantly lower in the multiligament group than in the isolated PCLR group at 5-year follow-up (*P* = .04). No significant differences were seen in the revision rates between the 2 groups at 2 and 5 years (3.9% and 4.6% for the isolated PCLR group and 1.7% and 1.9% for the multiligament group at 2 and 5 years, respectively) (Figure 2). No significant differences were found in revision rates between the groups at 5 years (4.6% and 1.9% for isolated PCL and multiligament injuries, respectively; *P* = .07). In a multivariate logistic regression analysis, the odds ratio for revision was 0.31 (95% CI, 0.01-0.97; *P* = .04) for the isolated PCLR group compared with the multiligament group as reference (Figure 2).

**Table 2:** Mean and Proportion of Patients With Subjective Failure (KOOS Quality of Life Subscale Score <44) at Various Assessment Points^*a*^

Assessment Point	Isolated PCLR (n = 185)	Multiligament Reconstruction (n = 446)	*P*
Preoperative	29 (90.1)	27 (85.7)	.24
2-year follow-up	50 (49.5)	50 (46.5)	.61
5-year follow-up	53 (46.7)	56 (34.2)	**.04**

a Data are expressed as mean score and proportion of patients with KOOS Quality of Life subscale score <44 (%). Boldface indicates statistical significance. KOOS was available in 359/631 (56.9%), 368/548 (67.2%), and 268/432 (62%) of the patients preoperatively and at 2- and 5-year follow-up, respectively. KOOS, Knee injury and Osteoarthritis Outcome Score; PCLR, posterior cruciate ligament reconstruction.

**Table 3:** KOOS Subscale Scores at Various Assessment Points^*a*^

KOOS Subscales	Isolated PCLR (n = 185)	Multiligament Reconstruction (n = 446)	*P*
Pain			.094
Preoperative	59 ± 17	63 ± 23	
2-year follow-up	69 ± 22	72 ± 22	
5-year follow-up	72 ± 21	76 ± 21	
Symptoms			.796
Preoperative	66 ± 17	66 ± 20	
2-year follow-up	65 ± 21	67 ± 20	
5-year follow-up	68 ± 21	71 ± 20	
Activities of Daily Living			.214
Preoperative	69 ± 19	66 ± 26	
2-year follow-up	78 ± 21	78 ± 23	
5-year follow-up	80 ± 19	82 ± 21	
Sport and Recreation			.047
Preoperative	30 ± 24	24 ± 25	
2-year follow-up	44 ± 29	40 ± 29	
5-year follow-up	46 ± 32	45 ± 29	
Quality of Life			.250
Preoperative	29 ± 15	27 ± 21	
2-year follow-up	50 ± 26	50 ± 25	
5-year follow-up	53 ± 26	56 ± 25	

a Values are expressed as mean ± SD. KOOS, Knee injury and Osteoarthritis Outcome Score; PCLR, posterior cruciate ligament reconstruction.

## Discussion

The most important finding of the current study was that there were high subjective failure rates after PCLR in both isolated and multiligament PCL injuries at both the 2- and the 5-year follow-up periods. Contrary to the hypothesis, the isolated and multiligament PCLR groups had no significant difference in their subjective failure rates at the 2-year follow-up, whereas isolated PCLR had a significantly higher subjective failure rate at 5-year follow-up versus the multiligament group. No significant differences were found in revision rates between the 2 groups at 2- or 5-year follow-up. We believe that this study demonstrates overall unsatisfactory short-term clinical results with PCLR at a national database level, and further studies are needed to fully investigate the potential causes of these unsatisfactory results.

This study found significant improvements in KOOS subscale scores after surgery for both the isolated and the multiligament PCLR groups. However, subjective failure of isolated PCLR (49.5%) and combined PCLR (46.5%) at 2 years was common. At 5 years, the subjective failure rates were similarly high (isolated 46.7%, multiligament 34.2%). Interestingly, the proportion of clinical failures decreased from 2- to 5-year follow-up points in the multiligament group. This may be explained by the prolonged rehabilitation time that is necessary after these major knee reconstructions. In the present study, it is worth noting that the multiligament population was a decade older than the patients with isolated PCL. It is possible that the expectations in the multiligament group were lower because of the extent of the injury and the patients’ age compared with the younger isolated PCL injury population. Overall, our findings are similar to those of many studies of other national or multisite registries.^16,21,22,25^ Studies have generally reported significant improvements after PCLR in comparison with presurgical levels.^16,21,22^ However, Lind et al^16^ evaluated the Danish Knee Ligament Reconstruction Registry at a mean 5-year follow-up and reported high rates of subjective failure, which they defined as KOOS QoL <44 (35% isolated PCLR and 45% multiligament PCLR) and revision surgery (3% isolated PCLR and 3.4% multiligament PCLR). In addition, 2 studies using the NKLR reported that patients undergoing isolated PCLR started at significantly lower baseline KOOS than patients undergoing isolated ACLR; further, those studies reported that isolated PCLR resulted in significantly improved KOOS scores versus presurgical levels and a similar overall mean improvement compared with isolated ACLR, although the patients who underwent ACLR reported higher KOOS levels after surgery given their higher starting KOOS scores.^1,22^

In the current study, the QoL subscale of the KOOS was selected as an indicator of subjective failure after PCLR. Previous studies have recommended using the KOOS QoL subscale to evaluate treatment effects after ACLR.^5,7,8^ The KOOS QoL subscale has been studied as an indicator of subjective failure after ACLR when the score is <44, which corresponds with a more than moderately decreased knee-related quality of life.^5,7^ In a study by Granan et al,^7^ a KOOS QoL score <44 was associated with 3.7 times higher risk of revision ACLR at a mean follow-up of 40 months. To our knowledge, the current study is the first time that this threshold has been applied in relation to PCL injuries. Using this definition for an inferior patient-reported outcome, we identified a subjective failure rate of 46.7% for isolated PCLR and 34.2% for combined PCLR injuries at 5-year follow-up. It is challenging to explain the reason for the high failure rates, but several factors that are not recorded in the registry may influence the results. If the registry had provided full data on graft diameter, bony morphology (decreased tibial slope),^2,3^ surgical technique (anatomic vs nonanatomic reconstructions; single- vs double-bundle PCLR),^24^ surgeon volume, and postoperative rehabilitation (weightbearing status; prone vs supine knee motion; dynamic bracing), all of which have been studied and noted to affect forces on PCL grafts,^11,15,23^ these data may have allowed the identification of subgroups that reported better results. Data on the influence of meniscal pathology in ACLR at short-term follow-up (2-5 years) are conflicting. Data from the NKLR have not shown meniscal pathology to play an important role in ACLR at short-term follow-up; however, no data exist on the effect of meniscal pathology and treatment in PCLR.^12^

Interestingly, the high subjective clinical failure rates could not be corroborated with the low overall revision PCLR rate. Even though revision rate is a hard endpoint, it is not reliable in evaluating outcomes after PCLR and multiligament surgery. In the present study, the revision rates were relatively low; however, functional outcomes based on KOOS QoL were not satisfactory and many patients had KOOS QoL <44, suggesting poor knee function. It is possible that many surgeons believe that improved objective surgical outcomes are less likely once a patient is evaluated for recurrent instability, leading to a high threshold to attempt revision PCLR. This study represents a real-world representation of PCLR outcomes in a national setting. This allowed us to compare groups of interest with a large number of patients included.

The main limitation of this study is the higher proportion of nonresponders for the patient-reported outcome data at 2-year (32.8%) and 5-year (38.0%) follow-up (see Table 2). In addition, the NKLR does not collect radiographic data. As such, this study was not able to objectively assess posterior tibial translation on PCL stress radiographs and correlate these objective data with any subjective clinical outcomes. Although our definition of subjective failure is suggested for ACLR patients, we believe it can be used for PCLR patients. However, cutoffs in patient-reported outcomes describing treatment outcomes are cohort-specific and should be defined in every cohort studied. Furthermore, we did not have data on graft diameter, surgical technique (anatomic vs nonanatomic reconstruction or single- vs double-bundle PCLR), surgeon volume, and postoperative rehabilitation (weightbearing status; prone vs supine knee motion; dynamic vs static bracing), which may affect outcomes. The registry does not collect data on patients’ osteoarthritis grade at baseline or surgeon experience, both of which may influence the outcomes.

## Conclusion

Patients who underwent PCLR had improved KOOS QoL scores relative to their preoperative state. However, the subjective failure rate was high for both isolated and multiligament PCLR. Within the first 2 years after reconstruction, patients who undergo isolated PCLR can be expected to have similar failure rates to patients who undergo combined ligament reconstructions.

## References

[1] Arøen A Sivertsen EA Owesen C Engebretsen L Granan LP . An isolated rupture of the posterior cruciate ligament results in reduced preoperative knee function in comparison with an anterior cruciate ligament injury. Knee Surg Sports Traumatol Arthrosc. 2013;21(5):1017-1022. 22801932 10.1007/s00167-012-2132-1

[2] Bernhardson AS Aman ZS DePhillipo NN , et al. Tibial slope and its effect on graft force in posterior cruciate ligament reconstructions. Am J Sports Med. 2019;47(5):1168-1174. 30896980 10.1177/0363546519827958

[3] Bernhardson AS DePhillipo NN Aman ZS , et al. Decreased posterior tibial slope does not affect postoperative posterior knee laxity after double-bundle posterior cruciate ligament reconstruction. Am J Sports Med. 2019;47(2):318-323. 30657698 10.1177/0363546518819786

[4] Chahla J Moatshe G Cinque ME , et al. Single-bundle and double-bundle posterior cruciate ligament reconstructions: a systematic review and meta-analysis of 441 patients at a minimum 2 years’ follow-up. Arthroscopy. 2017;33(11):2066-2080. 28866340 10.1016/j.arthro.2017.06.049

[5] Frobell RB Roos EM Roos HP Ranstam J Lohmander LS . A randomized trial of treatment for acute anterior cruciate ligament tears. N Engl J Med. 2010;363(4):331-342. 20660401 10.1056/NEJMoa0907797

[6] Granan LP Bahr R Steindal K Furnes O Engebretsen L . Development of a national cruciate ligament surgery registry: the Norwegian National Knee Ligament Registry. Am J Sports Med. 2008;36(2):308-315. 17989167 10.1177/0363546507308939

[7] Granan LP Baste V Engebretsen L Inacio MC . Associations between inadequate knee function detected by KOOS and prospective graft failure in an anterior cruciate ligament-reconstructed knee. Knee Surg Sports Traumatol Arthrosc. 2015;23(4):1135-1140. 24619491 10.1007/s00167-014-2925-5

[8] Ingelsrud LH Terwee CB Terluin B , et al. Meaningful change scores in the Knee injury and Osteoarthritis Outcome Score in patients undergoing anterior cruciate ligament reconstruction. Am J Sports Med. 2018;46(5):1120-1128. 29517924 10.1177/0363546518759543

[9] Kennedy NI LaPrade RF Goldsmith MT , et al. Posterior cruciate ligament graft fixation angles, part 1: biomechanical evaluation for anatomic single-bundle reconstruction. Am J Sports Med. 2014;42(10):2338-2345. 25091117 10.1177/0363546514541225

[10] Kennedy NI LaPrade RF Goldsmith MT , et al. Posterior cruciate ligament graft fixation angles, part 2: biomechanical evaluation for anatomic double-bundle reconstruction. Am J Sports Med. 2014;42(10):2346-2355. 25091116 10.1177/0363546514541226

[11] LaPrade CM Civitarese DM Rasmussen MT LaPrade RF . Emerging updates on the posterior cruciate ligament: a review of the current literature. Am J Sports Med. 2015;43(12):3077-3092. 25776184 10.1177/0363546515572770

[12] LaPrade CM Dornan GJ Granan LP LaPrade RF Engebretsen L . Outcomes after anterior cruciate ligament reconstruction using the Norwegian Knee Ligament Registry of 4691 patients: how does meniscal repair or resection affect short-term outcomes? Am J Sports Med. 2015;43(7):1591-1597. 25868635 10.1177/0363546515577364

[13] LaPrade RF Chahla J DePhillipo NN , et al. Single-stage multiple-ligament knee reconstructions for sports-related injuries: outcomes in 194 patients. Am J Sports Med. 2019;47(11):2563-2571. 31381372 10.1177/0363546519864539

[14] LaPrade RF Cinque ME Dornan GJ , et al. Double-bundle posterior cruciate ligament reconstruction in 100 patients at a mean 3 years’ follow-up: outcomes were comparable to anterior cruciate ligament reconstructions. Am J Sports Med. 2018;46(8):1809-1818. 29953296 10.1177/0363546517750855

[15] LaPrade RF Smith SD Wilson KJ Wijdicks CA . Quantification of functional brace forces for posterior cruciate ligament injuries on the knee joint: an in vivo investigation. Knee Surg Sports Traumatol Arthrosc. 2015;23(10):3070-3076. 25145947 10.1007/s00167-014-3238-4

[16] Lind M Nielsen TG Behrndtz K . Both isolated and multi-ligament posterior cruciate ligament reconstruction results in improved subjective outcome: results from the Danish Knee Ligament Reconstruction Registry. Knee Surg Sports Traumatol Arthrosc. 2018;26(4):1190-1196. 28547586 10.1007/s00167-017-4577-8

[17] Midttun E Andersen MT Engebretsen L , et al. Good validity in the Norwegian Knee Ligament Register: assessment of data quality for key variables in primary and revision cruciate ligament reconstructions from 2004 to 2013. BMC Musculoskelet Disord. 2022;23(1):231. 35264137 10.1186/s12891-022-05183-2 PMC8908681

[18] Noyes FR Barber-Westin SD . Posterior cruciate ligament revision reconstruction, part 1: causes of surgical failure in 52 consecutive operations. Am J Sports Med. 2005;33(5):646-654. 15722270 10.1177/0363546504271210

[19] Owesen C Aas E Årøen A . Surgical reconstruction is a cost-efficient treatment option for isolated PCL injuries. Knee Surg Sports Traumatol Arthrosc. 2018;26(4):1053-1058. 28710510 10.1007/s00167-017-4632-5 PMC5876254

[20] Owesen C Røtterud JH Engebretsen L Årøen A . Effect of activity at time of injury and concomitant ligament injuries on patient-reported outcome after posterior cruciate ligament reconstruction. Orthop J Sports Med. 2018;6(12):2325967118817297. 10.1177/2325967118817297 PMC6311657 30627591

[21] Owesen C Sandven-Thrane S Lind M , et al. Epidemiology of surgically treated posterior cruciate ligament injuries in Scandinavia. Knee Surg Sports Traumatol Arthrosc. 2017;25(8):2384-2391. 26387121 10.1007/s00167-015-3786-2 PMC5522502

[22] Owesen C Sivertsen EA Engebretsen L Granan LP Årøen A . Patients with isolated PCL injuries improve from surgery as much as patients with ACL injuries after 2 years. Orthop J Sports Med. 2015;3(8):2325967115599539. 10.1177/2325967115599539 PMC4622308 26535391

[23] Pierce CM O’Brien L Griffin LW LaPrade RF . Posterior cruciate ligament tears: functional and postoperative rehabilitation. Knee Surg Sports Traumatol Arthrosc. 2013;21(5):1071-1084. 22484415 10.1007/s00167-012-1970-1

[24] Schreier FJ Banovetz MT Rodriguez AN LaPrade RF . Cutting-edge posterior cruciate ligament reconstruction principles. Arch Bone Jt Surg. 2021;9(6):607-617. 35106325 10.22038/ABJS.2021.59467.2946 PMC8765199

[25] Tucker CJ Cotter EJ Waterman BR , et al. Functional outcomes after isolated and combined posterior cruciate ligament reconstruction in a military population. Orthop J Sports Med. 2019;7(10):2325967119875139. 10.1177/2325967119875139 PMC6791041 31656823

[26] Ulstein S Årøen A Engebretsen L , et al. Effect of concomitant cartilage lesions on patient-reported outcomes after anterior cruciate ligament reconstruction: a nationwide cohort study from Norway and Sweden of 8470 patients with 5-year follow-up. Orthop J Sports Med. 2018;6(7):2325967118786219. 10.1177/2325967118786219 PMC6058423 30057926

[27] von Elm E Altman DG Egger M , et al. The Strengthening the Reporting of Observational Studies in Epidemiology (STROBE) statement: guidelines for reporting observational studies. Int J Surg. 2014; 12(12):1495-1499. 25046131 10.1016/j.ijsu.2014.07.013

[28] Winkler PW Zsidai B Wagala NN , et al. Evolving evidence in the treatment of primary and recurrent posterior cruciate ligament injuries, part 2: surgical techniques, outcomes and rehabilitation. Knee Surg Sports Traumatol Arthrosc. 2021;29(3):682-693. 33125531 10.1007/s00167-020-06337-2 PMC7917042

[29] Yoon KH Bae DK Song SJ Cho HJ Lee JH . A prospective randomized study comparing arthroscopic single-bundle and double-bundle posterior cruciate ligament reconstructions preserving remnant fibers. Am J Sports Med. 2011;39(3):474-480. 21098819 10.1177/0363546510382206

[30] Yoon KH Kim EJ Kwon YB Kim SG . Minimum 10-year results of single- versus double-bundle posterior cruciate ligament reconstruction: clinical, radiologic, and survivorship outcomes. Am J Sports Med. 2019;47(4):822-827. 30753096 10.1177/0363546518825257

[31] Ytterstad K Granan LP Ytterstad B , et al. Registration rate in the Norwegian Cruciate Ligament Register: large-volume hospitals perform better. Acta Orthop. 2012;83(2):174-178. 22489890 10.3109/17453674.2012.678800 PMC3339533

